# Flow-Volume Loop: A Tool for Evaluating the Position of the Laryngeal Mask Airway Intraoperatively

**DOI:** 10.7759/cureus.73652

**Published:** 2024-11-13

**Authors:** Maria Zozefin Nikolopoulou, Stamatios Katsimperis, Dionisia Nikolaki, Maria Kokolaki

**Affiliations:** 1 Department of Anaesthesiology, Sismanogleio General Hospital, Athens, GRC; 2 Second Department of Urology, National and Kapodistrian University of Athens, Sismanogleio General Hospital, Athens, GRC

**Keywords:** airway, flow-volume loop, laryngeal mask airway (lma), mechanical ventilation, obstruction

## Abstract

The flow-volume loop (FVL) is a valuable yet often underutilized tool for assessing the placement of the laryngeal mask airway (LMA) during surgery. It provides real-time graphical data on airway patency and ventilation. These cases examine the role of FVL in identifying LMA misplacement. Abnormal patterns, such as truncated expiratory curves and reduced peak flows, can indicate partial obstructions that might otherwise go unnoticed. By monitoring FVL, clinicians can quickly detect and correct misalignment, thereby enhancing patient safety and ventilator effectiveness during surgical procedures.

## Introduction

In recent years, the use of laryngeal mask airway (LMA) devices has become increasingly popular among practitioners performing elective surgeries. This rise in popularity can be attributed to several factors, including minimal irritation or injury to the airway and the fact that the administration of a muscle relaxant is not necessary for insertion [1,2]. While there are multiple methods to confirm the successful placement of the LMA, verifying proper placement can sometimes be challenging. The flow-volume loop is a graphical representation of inspiratory and expiratory flow for a specific tidal volume. It aids in the differential diagnosis of various pulmonary pathologies and airway obstructions. Although the flow-volume loop is not commonly used intraoperatively during mechanical ventilation, it can provide valuable information regarding the patient's airway patency and ventilation ​[3]. By carefully interpreting the loop, clinicians can adjust the ventilator settings to optimize oxygenation, ventilation, and patient safety. For this reason, we aimed to assess the flow-volume loop's potential as an intraoperative tool for identifying misplacement of the LMA, ensuring optimal airway management. 

## Case presentation

Our department routinely uses a laryngeal mask airway (LMA) for brief elective surgeries, provided there are no significant contraindications, such as decreased pulmonary compliance, high airway resistance, oropharyngeal pathology, risk for aspiration, or airway obstruction. Since the flow volume loop serves as an excellent diagnostic tool and indicates airway obstruction, we decided to configure the ventilator screen to display the flow volume loop whenever an LMA is inserted. Our purpose was to observe whether the loop showed any obstruction caused by the insertion of the LMA.

After a period of three months of applying this method, we identified two cases of laryngeal mask misplacement, which we detected using a flow-volume loop. In both cases, undergoing ureterolithotripsy, American Society of Anesthesiologists (ASA) standard monitoring was set up, including non-invasive blood pressure, electrocardiography, pulse oximetry, end-tidal CO_2,_ and intravenous access was established. Patients were induced under general anesthesia using propofol 1% at a dose of 3 mg/kg, fentanyl at 1 μg/kg, and rocuronium at 1 mg/kg, and maintained on sevoflurane. The parameters for mechanical ventilation were established as follows: volume control ventilation was set with a tidal volume of 7 ml/kg, a respiratory rate of 12-16 breaths per minute, and a positive end-expiratory pressure (PEEP) of 5 cm H_2_O. The second-generation LMA size for each case was selected based on the patient's ideal body weight. The correct position was also confirmed by auscultating the hemithoraces for breathing sounds and the thyroid cartilage for potential sealing leaks and the presence of a capnography waveform. 

Case 1

A 53-year-old male patient was scheduled for elective ureterolithotripsy. The patient had a medical history of hypertension and type 2 diabetes mellitus, for which he was receiving pharmacological treatment. He had no previous history of surgical procedures or hospitalizations. Preoperative hematological and biochemical laboratory results were within normal ranges. An airway evaluation indicated a Mallampati score of 2, a mouth opening greater than 3 cm, a thyromental distance of 6 cm, and good neck mobility.

**Figure 1:**
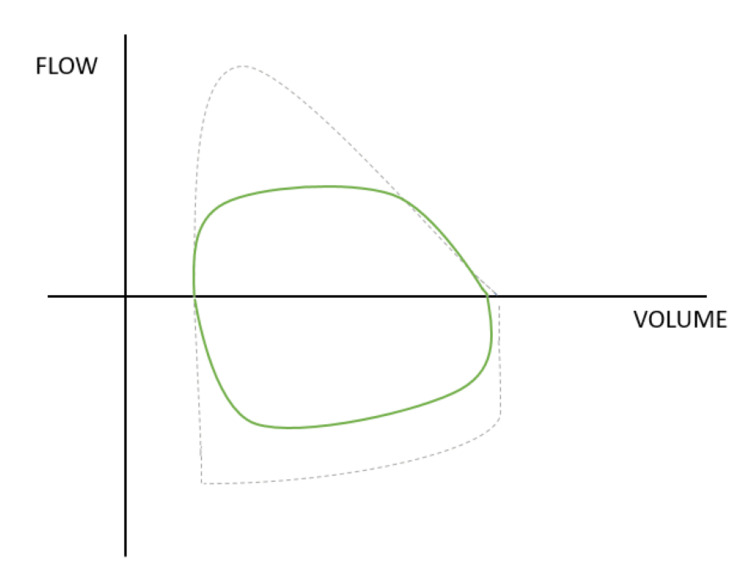
Flow-volume loop of a fixed upper airway obstraction by LMA X-axis: volume in milliliters, Y-axis: volume in milliliters/minute, expiration: above the X-axis, inspiration: below the X-axis, dashed line: a normal flow-volume loop, green line: abnormal flow-volume loop due to obstruction by the LMA, LMA: laryngeal mask airway. The flow-volume loop is drawn by us, attempting to accurately depict how the loop we observed was structured.

During the first case, following the insertion of the laryngeal mask airway (LMA) and its connection to the ventilator, the flow volume loop displayed a truncated flattened expiratory curve and a rounded inspiratory curve, along with reduced peak expiratory and inspiratory flows (see Figure 1). This presentation resembled that of a fixed upper airway obstruction, which in these instances was caused by the malposition of the LMA. Additionally, we observed an increase in peak inspiratory pressure (PIP) and an almost absent capnography waveform. We reinserted the LMA using a different maneuver, resulting in the correction of the flow-volume loop. The correct position was also confirmed by auscultation and the presence of a capnography waveform.

Case 2

A 46-year-old male, scheduled for elective ureterolithotripsy, presented to us. His medical history included thalassemia major for which he was being regularly transfused. He also had paroxysmal atrial fibrillation, Hashimoto thyroiditis under medical management, and osteoporosis. He had a history of two previous ureterolithotripsies. His spirometry showed mild to moderate restrictive pulmonary disease, possibly due to thalassemia. Lab tests were within normal ranges. Upon airway examination, he had a Mallampati 3, mouth opening greater than 3 cm, thyromental distance was 6 cm with good neck mobility.

**Figure 2:**
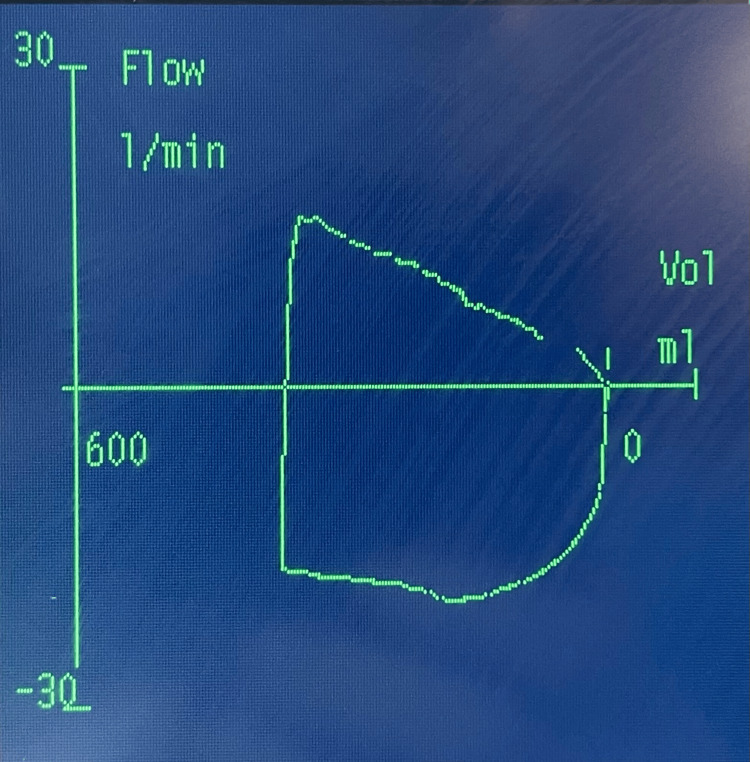
Flow-volume loop of a partial airway obstruction caused by the LMA x-axis: volume in milliliters, y-axis: volume in milliliters/minute expiration: above the x-axis, inspiration: below the x-axis reduced peak expiratory flow and a slightly rounded inspiratory curve with a diminished flattened peak inspiratory flow

**Figure 3:**
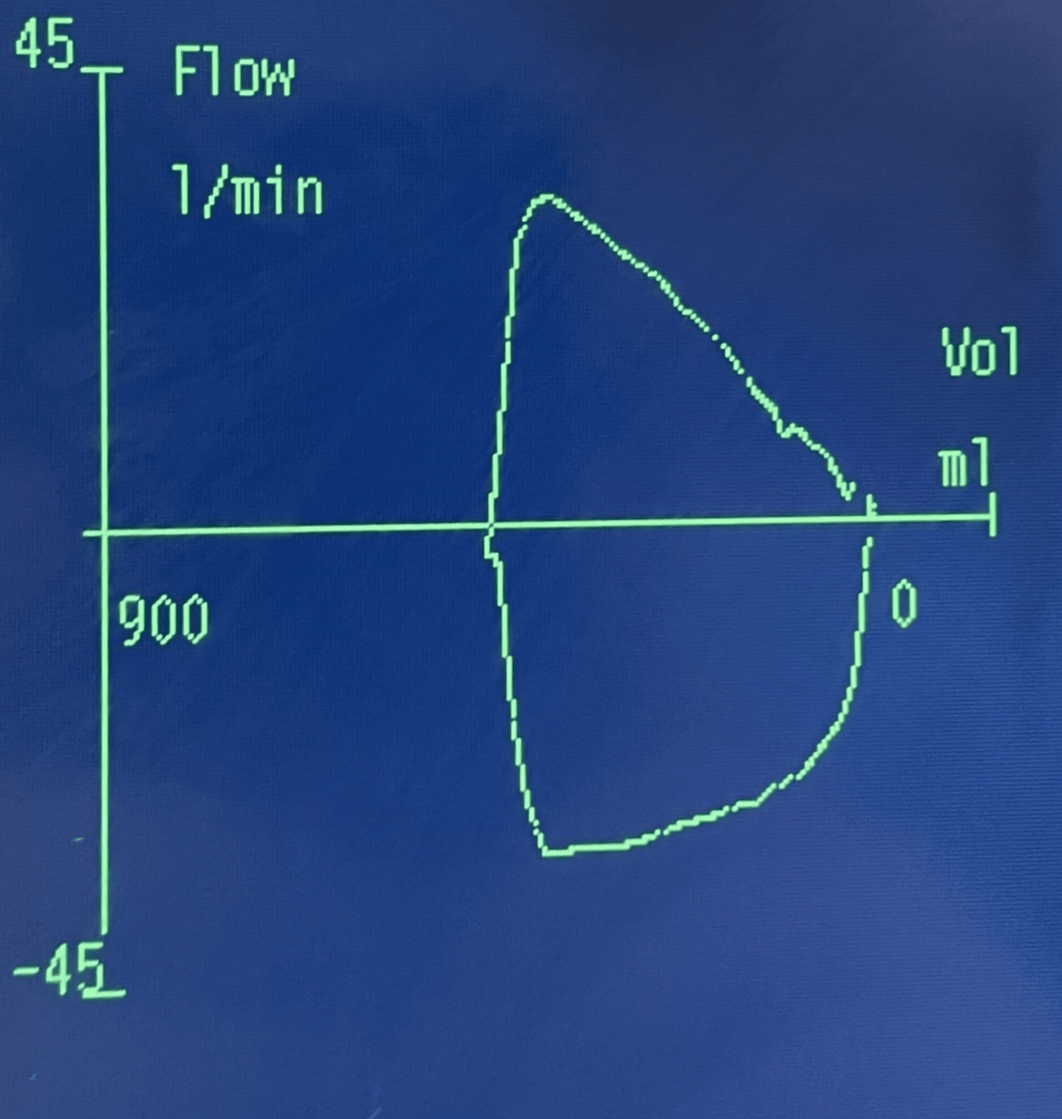
Normal flow-volume loop after the correction of the LMA position

The second case involved a patient with mild to moderate restrictive pulmonary disease due to thalassemia major. After the LMA was inserted, a standard assessment was conducted to confirm proper placement. The capnography waveform was normal, with normal breath sounds on both sides of the chest. After some seconds, the peak inspiratory pressure (PIP) was raised, which was attributed to the patient's underlying pathology. We adjusted the setup to pressure control ventilation but still struggled to deliver the desired tidal volume at low pressures. Despite these challenges, the patient remained hemodynamically stable, with adequate oxygenation and a SpO2 of 99%. We attempted to pass a nasogastric tube, but it could not progress beyond a certain point. This led us to suspect that the LMA was not properly positioned, which, combined with the other findings, was likely causing a partial obstruction. The flow-volume loop showed nearly normal expiration, but there was a reduced peak expiratory flow and a slightly rounded inspiratory curve with a diminished peak inspiratory flow (see Figure 2). After performing some maneuvers, withdrawing the LMA about 5cm and reinserting it, the nasogastric tube was successfully passed, and drainage of gastric content confirmed its position. The morphology of the flow-volume loop improved, and we were able to ventilate the patient in volume control mode (see Figure 3). 

## Discussion

Malposition of LMA may lead to partial or complete airway obstruction ​[4]. Some of the most common causes of obstructions caused by LMAs are the downfolding of the epiglottis within the bowl of the LMA and the distal part of the LMA itself, blocking the laryngeal inlet ​[5,6]​. Depending on the degree of the obstruction, the findings can vary. They may range from lower inspiratory and expiratory peak flows and truncated curves to more significant issues, such as increased peak inspiratory flow and an inability to ventilate ​[7]​.

In the first case, the morphology of the loop was characteristic of fixed upper airway obstruction, evidenced by a truncated and flattened peak expiratory flow curve, as well as a similarly truncated peak inspiratory flow curve. It's important to note that an incomplete obstruction may not always have a disruptive effect.

In the second case, the loop was almost normal, making it difficult for us to evaluate these changes as a partial obstruction. It was also demonstrated that passing a nasogastric tube can be very helpful in confirming the position of the LMA, a practice that is not always done [8]. This led us, then, to the suspicion that the previously mentioned finding was not due to his pathology but rather due to malposition causing a partial obstruction, something already detected by the loop. A flow volume loop is not only valuable for diagnosing pulmonary conditions. In a patient under general anesthesia who is on mechanical ventilation, the loop can help identify not only obstructions, as in our cases, but also issues such as secretions, cuff leaks, and air trapping ​[9]​.

As a real-time representation of airflow, it can provide more detailed information about the cause of increased airway pressure, helping us differentiate between causes​ [10]. Learning to interpret the flow volume loop is generally intuitive, as the curves are characteristic for each possible case. These cases indicate that using flow-volume loop monitoring can help detect LMA mispositioning early, enhancing airway management and patient safety during surgery. It can also offer the anesthesiologist important information about the patient's ventilation, making it a valuable tool. 

## Conclusions

The flow-volume loop, while commonly employed in pulmonary diagnostics, is not often used during surgical procedures. It can graphically represent airway resistance and flow patterns, offering a valuable means to identify subtle airway obstructions that may arise from improper positioning. However, it should not be relied upon as the primary or only method for confirming LMA placement.
